# New therapeutics for soft tissue sarcomas: Overview of current immunotherapy and future directions of soft tissue sarcomas

**DOI:** 10.3389/fonc.2023.1150765

**Published:** 2023-03-14

**Authors:** Gyuhee Seong, Sandra P. D’Angelo

**Affiliations:** ^1^ Department of Medicine, SUNY Downstate Health Sciences University, Brooklyn, NY, United States; ^2^ Department of Medicine, Memorial Sloan Kettering Cancer Center and Weill Cornell Medical College, New York, NY, United States

**Keywords:** soft tissue sarcoma, immune checkpoint inhibitor, adoptive immunotherapy, cancer testis antigen, T-cell receptor therapy, chimeric antigen receptor (CAR) T-cell, tumor-infiltrating lymphocyte, tumor microenvironment

## Abstract

Soft tissue sarcoma is a rare and aggressive disease with a 40 to 50% metastasis rate. The limited efficacy of traditional approaches with surgery, radiation, and chemotherapy has prompted research in novel immunotherapy for soft tissue sarcoma. Immune checkpoint inhibitors such as anti-CTLA-4 and PD-1 therapies in STS have demonstrated histologic-specific responses. Some combinations of immunotherapy with chemotherapy, TKI, and radiation were effective. STS is considered a ‘cold’, non-inflamed tumor. Adoptive cell therapies are actively investigated in STS to enhance immune response. Genetically modified T-cell receptor therapy targeting cancer testis antigens such as NY-ESO-1 and MAGE-A4 demonstrated durable responses, especially in synovial sarcoma. Two early HER2-CAR T-cell trials have achieved stable disease in some patients. In the future, CAR-T cell therapies will find more specific targets in STS with a reliable response. Early recognition of T-cell induced cytokine release syndrome is crucial, which can be alleviated by immunosuppression such as steroids. Further understanding of the immune subtypes and biomarkers will promote the advancement of soft tissue sarcoma treatment.

## 1 Introduction

Sarcomas are a rare and heterogeneous group of solid tumors of mesenchymal origin, accounting for only 1% of all adult malignancies. They can be divided broadly into soft tissue sarcomas (STS), which originate in the fat, muscle, nerve, nerve sheath, blood vessels, and other connective tissues or the bone.

More than 70 different histologic subtypes of STS have been identified (1). Soft tissue sarcoma is an aggressive disease with a 40 to 50% metastasis rate, with a 5-year survival rate of 30%. STS most commonly metastasizes to the lungs; tumors in the abdominal cavity more commonly metastasize to the liver and peritoneum (2).

The limited durable response with traditional surgery, radiation, and chemotherapy in advanced-stage sarcoma has prompted research in novel immunotherapy of soft tissue sarcoma.

### 1.1 Immune microenvironment of sarcoma

The tumor microenvironment (TME) comprises a tumor, stromal cells, and immune cells such as macrophages, lymphocytes, and extracellular matrix (3). Tumor cells take advantage of TME over time, and genetic/epigenetic changes of the tumor and rearrangement of TME are pivotal in tumorigenesis (4).

Tumor associated macrophages (TAMs) are distinguished components in TME. Tumors secrete high levels of colony-stimulating factor 1 (CSF-1), which converts M1 macrophage (classically activated, tumoricidal) to M2 macrophage/TAMs (alternatively activated, tumor-promoting) and stimulates tumor growth and metastasis along with CCL2 (5).

Sarcoma is traditionally considered an immunologically quiet tumor with low tumor mutational burden (1.06 mutations/Mb) and immunosuppressive TME (high levels of hypoxia-inducible factor 1 α (HIF1α), macrophages, neutrophils, and decreased T-cell levels) (6). A subset of sarcomas are sensitive to ICIs. They are ‘hot’/immune-sensitive tumors with high TMB, interferon, CD8 lymphocytes, and PD-L1 expression (7, 8).

A very recent paper highlights the significant prognostic value of systemic inflammatory indexes as a prognostic marker in terms of PFS and OS in STS patients who progressed on anthracycline. A low lymphocyte-to-monocyte ratio (LMR) was associated with worse OS (p = 0.006). Interestingly, low lymphocyte-to-monocyte ratio (LMR) was an indicator of trabectedin efficacy, which could be applied in clinical practice (9). In a previous study in 2021, 3D-cultured cells from leiomyosarcoma and undifferentiated pleomorphic sarcoma (UPS) surgical specimens were treated with trabectedin and demonstrated the involvement of ECM-associated genes such as *mmps* and their inhibitor *timp1*, emphasizing the potential role of ECM in the activity of trabectedin (10).

It was proposed that tumors with high PD-1 expression and tumor-infiltrating lymphocytes (TILs) respond well to ICIs (11). Sarcomas have relatively low PD-1 and TILs. Various studies have revealed conflicting results regarding how PD1 expression impacts prognosis. A recent review of Phase II trials demonstrated that 30% of patients with PD-L1 expression (≥1%) achieved a response. However, 7% of PD-L1 negative patients also achieved a response, underscoring the limitation of PD-L1 as a prognostic marker (12). A subsequent analysis of SARC028 revealed that higher TILs at baseline were associated with a better PFS.

In this article, we will review current immunotherapy of soft tissue sarcoma, highlighting prominent trials with immune checkpoint inhibitors and adoptive cellular therapies, including engineered T-cell receptor targeting cancer testis antigens (CTA), chimeric antigen receptor (CAR) T-cell therapies and tumor-infiltrating lymphocytes (TILs).

## 2 Immune checkpoint inhibitors

Immune checkpoint inhibitors (ICI) regulate critical inhibitory signals of T-cells such as PD-1/PD-L1 and CTLA-4 axes as monotherapy or in combination with chemotherapy. ICIs are FDA-approved to treat more than 50 cancer types, including advanced solid tumors, MMR-deficient tumors, and tumors with a high tumor mutation burden (13).

SARC028 was a significant Phase II trial published in 2017, which first demonstrated the efficacy of pembrolizumab (PD-1 inhibitor) in some STS, notably in undifferentiated pleomorphic sarcoma (UPS) (4 of 10) and dedifferentiated liposarcoma (dLPS) (2 of 10) (14). The final results of SARC028 expansion cohorts confirmed effectiveness in UPS, with an objective response rate (ORR) of 23%, but not in dedifferentiated/pleomorphic liposarcoma (LPS) with an ORR of 10% (15).

In the Phase II Alliance A091401 trial, patients with metastatic sarcoma were treated with nivolumab (PD-1 inhibitor) with or without ipilimumab (CTLA-4 inhibitor). Dual immune checkpoint blockade demonstrated an overall response (ORR) of 16%. Responses were confirmed in leiomyosarcoma (uterine (n=1), non-uterine (n=1)), myxofibrosarcoma (n=1), UPS (n=2), and angiosarcoma (n=1) (16). In a phase II study for advanced uterine leiomyosarcoma, none of the 12 patients responded to nivolumab alone (17). In a subsequent Phase II expansion cohort study, combination therapy of nivolumab and ipilimumab resulted in an ORR of 28.6% in UPS and 14.3% in dedifferentiated liposarcoma (18). In a DART trial by SWOG, a phase II trial of ipilimumab and nivolumab in angiosarcoma demonstrated an ORR of 25% (19). On December 2022, atezolizumab was granted FDA approval for unresectable or metastatic alveolar soft part sarcoma (ASPS) (ORR = 24%, NCT03141684).

Myxofibrosarcoma (MFS) expresses high levels of immune microenvironment markers, and some case reports support PD-1 inhibition in myxofibrosarcoma, which is further explored in a Phase II trial (ENVASARC, NCT04480502) (20–23).

ICI response in soft tissue sarcoma has been modest and histologic-specific, especially in UPS, dLPS, ASPS, and angiosarcoma.

### 2.1 ICI and local/systemic therapy

Combinational strategies with ICI and local/systemic therapies can overcome soft tissue sarcoma resistance mechanisms. Local therapies to complement ICI consist of isolated limb infusion and radiation.

Isolated limb infusion (ILI) is a minimally invasive administration of high-dose chemotherapy to treat STS in the extremities (24). Two patients with recurrent myxofibrosarcoma responded to melphalan *via* ILI and pembrolizumab (1=partial response, 1=complete response) (25). This promising case prompted a subsequent Phase II trial with pembrolizumab plus the infusion of melphalan and dactinomycin (NCT04332874).

Radiation therapy is another local therapy to activate anti-tumor immunogenicity in the tumor microenvironment through the cGAS-STING pathway and subsequent CD8+ T cell activation (26, 27). There are approximately ten ongoing trials to investigate the effect of radiation in addition to ICI.

Chemotherapy enhances immunosurveillance by releasing type I interferon (IFN), and increasing M2 macrophages, CD8+ T cells, and NK cells in a tumor microenvironment (28, 29).

Two Phase II trials of doxorubicin and pembrolizumab from Pollack et al. and Livingston et al. demonstrated promising ORR of 19% in advanced sarcoma and 36.7% in advanced STS, respectively (30, 31). In a Pollack et al. study, grade 3+ treatment-related adverse effects (TRAEs) such as neutropenia (6/37), leukopenia (1/37), and febrile neutropenia (1/37), heart failure due to doxorubicin (2/37), and adrenal insufficiency (1/37) and hypothyroidism (7/37) due to pembrolizumab were observed. In a Livingston et al. study, grade 3+ TRAEs include neutropenia and leukopenia (11/30 each), and anemia (8/30). Arthralgia (3/30), fatigue (2/30), autoimmune disorder (2/30), and increased lipase (2/30) were grade 3+ TRAEs attributed to pembrolizumab. Additionally, pembrolizumab-related synovitis/myositis (n=1), autoimmune hepatitis (n=1), and autoimmune nephritis (n=1) were observed, and all patients responded to steroids. Grade 5 adverse events were not reported in both studies.

Trabectedin, in addition to ipilimumab and nivolumab, revealed an ORR of 19.5% in metastatic STS (32). Grade 4 adverse events include anemia, neutropenia, thrombocytopenia, and increased AST/ALT and CPK. Grade 5 rhabdomyolysis was observed in one patient.

Another strategy to augment immune response in STS is to combine small molecule inhibitors such as tyrosine kinase inhibitors (TKI). In the Phase II Immunosarc trial, TKI sunitinib with nivolumab in metastatic or locally advanced STS led to an ORR of 21%, with 48% of 6-month PFS (33). Wilky et al. demonstrated the efficacy of Axitinib (VEGF receptor TKI) and pembrolizumab in advanced sarcoma. None achieved a complete response. 8 out of 32 patients achieved a partial response (ORR 25.0%), with most responses occurring in ASPS (6/11, ORR 54.5%) (34).

Pembrolizumab is FDA-approved in many cancers such as advanced melanoma, Merkel Cell Carcinoma, Cutaneous Squamous Cell Carcinoma, and non-small cell lung cancer, either alone or with other therapies (35–38).

Phase II trials combining systemic therapy with pembrolizumab in sarcoma are in progress: Pembrolizumab + eribulin (NCT03899805), pembrolizumab + gemcitabine (NCT03123276), pembrolizumab + lenvatinib (NCT04784247), pembrolizumab + doxorubicin (NCT03056001), pembroliumab + cabozantinib (PEMBROCABOSARC, NCT05182164), pembrolizumab + epacadostat (IDO1 Inhibitor)(NCT03414229).

Other PD-1 inhibitors in sarcoma are investigated in Phase II trials. Nivolumab + Gemcitabine/Doxorubicin/Docetaxel (GALLANT, NCT04535713), Retifanlimab (PD-1 inhibitor) + Gemcitabine/Docetaxel (NCT04577014), Sintilimab (PD-1 inhibitor) + Doxorubicin/Ifosfamide (NCT04356872) and Camrelizumab (PD-1 inhibitor) + Doxorubicin/Ifosfamide (NCT04606108) are in progress.

Future research should aim to identify biomarkers in STS to augment responses of ICI with and without local/systemic therapies in each patient.

## 3 Adoptive cellular therapies

Successful T-cell treatments for hematological malignancies have sparked interest in researching T-cell therapies for solid tumors such as sarcomas.

One of sarcoma’s primary immune evasion strategies is inadequate neoantigens/antigen recognition, which fails to create enough tumor-specific T cells and immune responses. Adoptive cellular therapies hope to avoid this phase by supplying a significant amount of autologous T cells specifically designed for a particular antigen. Autologous T cells are obtained from peripheral blood or the original tumor and then amplified. Potential approaches include engineered T-cell receptor (TCR) and chimeric antigen receptor (CAR) T-cell therapy and tumor-infiltrating lymphocyte (TIL) therapy with sarcoma.

### 3.1 Engineered T-cell receptor therapy

Cancer testis antigens (CTA) are tumor-associated antigens (TAA) that are typically present in fetal development (placenta and embryo) or at immune-privileged sites without MHC class I (testes) (39). Sarcomas express higher than normal CTAs, especially in SS and myxoid/round cell liposarcoma (40, 41). Sarcomas express a variety of CTAs such as the NY-ESO-1, MAGE, and GAGE family and fetal acetylcholine receptors (42).

NY-ESO-1 and MAGE family are intracellular antigens that must be processed and presented with MHC. TCR T cells require patients with matching HLA allele subtypes, often HLA-A2, which compose approximately 30% of the population. Modified TCR T cells recognize processed peptides *via* HLA-A2-specific manner and mount immune responses (43).

In 2011, Robbins et al. successfully investigated the antitumor response of NY-ESO-1-specific TCRs with high dose interleukin-2 in refractory synovial sarcoma (SS). Objective clinical responses were observed in 4 of 6 SS patients. A partial response lasted for 18 months in a patient with synovial sarcoma (44). Long-term follow-up study which enrolled 12 additional SS patients, revealed that 11 of 18 patients with SS who received anti-NY-ESO-1 TCRs responded to therapy (61%), and one had a complete response (45).

In a Phase I trial in 2018, T cells expressing NY-ESO-1c259 (Letetresgene autoleucel), a modified TCR recognizing NY-ESO-1/LAGE1a peptide, demonstrated an ORR of 50% (6/12) in metastatic SS following a lymphodepleting regimen of fludarabine and cyclophosphamide. Remarkably, self-generating pools of NY-ESO-1c259T cells persisted *in vivo* for at least 6 months in all patients who responded. No fatal adverse events were reported. Grade 3-4 adverse events include lymphopenia, leukopenia, neutropenia, anemia, thrombocytopenia, and hypophosphatemia. Cytokine release syndrome was reported in five patients, with median onset within 4 days and a median duration of 10 days (46).

High dose fludarabine-containing regimen is necessary for the efficacy of NY-ESO-1c259 TCR, likely correlated with elevated IL-7 and IL-15, and TAM modulation (47).

Afamitresgene autoleucel (ADP-A2M4 SPEAR TCRs directed against the MAGE-A4) revealed comparable efficacy. Phase I study with MAGE-A4c1032 TCR by Hong et al. observed an ORR of 25% in advanced solid tumors, and all partial responses were in patients with synovial sarcoma. Two patients had trial-related deaths due to aplastic anemia and CVA (48). A subsequent phase II study with afamitresgene autoleucel revealed an ORR of 40% in 25 patients with a tolerable safety profile in advanced/metastatic SS and Myxoid/Round Cell Liposarcoma (MRCLS) (49).

Although engineered TCR in advanced soft tissue sarcoma presents promising efficacy, there are some limitations to overcome, particularly the HLA-A2 requirement, manufacturing timelines/cost, and associated toxicities such as cytokine release syndrome. Furthermore, there are heterogenous CTA expressions in different types of sarcomas, and broad applicability may be limited (43).

### 3.2 Chimeric antigen receptor T-cell therapies

CARs are chimeric antigen receptors artificially engineered to recognize naturally occurring tumor surface antigens and activate T-cells in an MHC-independent manner (50).

C19-targeted CAR T-cell therapies for hematologic malignancies such as CD19-positive B-cell acute lymphoblastic leukemia and B-cell lymphomas have been successful. In 2022, Ciltacabtagene autoleucel, B-cell maturation antigen-directed CAR T-cell, was FDA-approved for patients with refractory or relapsed multiple myeloma who received at least four lines of therapy (CARTITUDE-1, NCT03548207). Further efforts to expand CAR T-cell therapies in solid tumors are ongoing but have not shown major significance yet.

In Phase I/II trial in HER2-positive sarcomas, including 16 osteosarcomas, one Ewing sarcoma, one primitive neuroectodermal tumor, and one desmoplastic small round cell tumor, HER2-CAR T cell therapy induced stable disease in four patients without significant toxicity (51).

In another Phase I trial, ten HER2+ refractory/metastatic patients (osteosarcoma (5), rhabdomyosarcoma (3), Ewing sarcoma (1), and synovial sarcoma (1)) were enrolled and treated with HER2-CAR T cells and lymphodepletion with either fludarabine or in combination with cyclophosphamide. At the initial follow-up at 6 weeks, 4 patients had progression, and 4 patients achieved stable disease. Overall survival at 1 year was 60% for patients treated with HER2-CAR T cells and lymphodepletion (52).

EGFR, GD2, insulin-like growth factor 1 receptor (IGF-1R), tyrosine kinase orphan-like receptor 1 (ROR1), CD44v6, and NK cell activating receptor group 2-member D (NKG2D) are potential targets in sarcoma, and early phase trials are underway to investigate the efficacy of CAR therapies for these targets.

CAR T-cell therapies will have to overcome a few obstacles in the future. CAR T-cell therapies have limited cancer-specific antigens, whereas TCRs recognize peptides presented *via* MHC class 1, which essentially include whole proteasome (53, 54). Until now, CAR-T therapies seek more specific targets in solid tumors, which are conserved and do not convey toxicity to healthy tissue, to improve long-term efficacy (55).

Cytokine release syndrome (CRS) is one of the adverse effects of both TCR and CAR T-cell therapy following T-cell administration. CRS is an acute, systemic response from immune stimulation in an “on-target and on-tumor” manner. T-cell therapies can also induce unexpected “on-target, off-tumor” autoimmunity, which damages healthy cells by recognizing shared antigens (56–58). It is crucial to promptly recognize and treat immune-mediated adverse effects, which can be alleviated by immunosuppression such as Tocilizumab and steroids if needed.

### 3.3 Tumor-infiltrating lymphocytes therapies

Tumor-infiltrating lymphocytes (TIL) are extracted from tumors and administered to the patients after *ex vivo* expansion (59, 60). TIL had reproducible effects in melanoma. In a phase 3 trial by Rohaan et al. in 2022, TIL therapy demonstrated an ORR of 49% (41/84) in advanced melanoma (61). There has not yet demonstrated satisfactory efficacy in other solid tumors.

In 2021, Mullinax et al. investigated a rapid expansion protocol that TIL cultures from soft tissue sarcoma resection can expand enough for clinical adoptive cell therapy, which led to an ongoing Phase I trial (NCT04052334) (62).

Current challenges for TIL therapies include high cost due to the personalized nature of TIL therapies, and toxicities from high-dose IL-2, which is given post-TIL administration (63, 64).

## 4 Cancer vaccines

Talimogene laherparepvec (T-VEC) is an oncolytic viral immunotherapy *via* intratumor injection. It enhances immunogenicity *via* antigen presentation and tumor-specific T cells. T-VEC is the first viral immunotherapy approved for metastatic melanoma (65).

In a Phase II trial, 20 patients with advanced/metastatic sarcoma were treated with an oncolytic virus, T-VEC, with pembrolizumab, which demonstrated an ORR of 35% and a median duration of response of 56.1 weeks (66).

Vaccine therapies have been explored for decades without satisfactory results, likely due to suppressive tumor microenvironment. Current efforts are utilizing novel vectors to promote specificity and strength of immune response.

A novel study by Somaiah et al. demonstrated the efficacy of LV305, a lentivirus vector designed to induce NY-ESO-1 in dendritic cells *in vivo*, improving immune response against tumor cells (67). ORR was 4.2% in sarcoma (1/24 in SS).

CMB305 (a heterologous vaccine for NY-ESO-1 and TLR 4 agonist) is a good vehicle for synovial sarcoma and myxoid/round cell liposarcoma patients, and it was subsequently assessed in a Phase Ib study (68, 69). The study demonstrated a disease control rate of 61.9% and OS of 26.2 months in 64 sarcoma patients. Phase II study with CMB305 and atezolizumab (PD-L1 antibody) compared to atezolizumab alone in STS did not reveal significant improvement in PFS or OS compared to atezolizumab alone (70).

## 5 Future directions

Although adoptive cellular therapies offer potential individual treatments, they are still in their infancy for soft tissue sarcoma. Targeting fusion-derived cancer testis antigens such as NYESO-1 and MAGEA-4 has shown benefits in limited sarcomas such as synovial sarcoma and Myxoid/Round Cell Liposarcoma (71–73).

Colony-stimulating factor-1 (CSF1) promotes “macrophage polarization”, increasing M2/M1 macrophage ratio. CSF1R inhibitor can be a potent immunomodulator by prohibiting the recruitment of TAMs into TME (74). CSF1R-targeting agents have shown a relatively tolerable safety profile but only modest clinical activity.

TTI-621 is a recombinant fusion antibody for SIRPα, a binding domain for CD47, which interrupts inhibition of macrophage phagocytosis mediated by CD47 and stimulates phagocytosis. Combination of doxorubicin with TTI-621 (anti-CD47 antibody) has shown anti-tumor effect in animal models, especially in tumors which express high number of CD47 and macrophages, such as leiomyosarcoma (75). Phase I/II study with TTI-621 alone and in combination with doxorubicin for patients with advanced leiomyosarcoma is underway (NCT04996004).

DR5 Agonist Antibody targeting the TRAIL-TNF axis, which promotes tumor-specific apoptosis, is evaluated in a Phase II study of chondrosarcomas (NCT04950075). NK cell therapies have limited data in solid tumors, and trials for sarcoma (NCT01875601, NCT02890758, NCT03420963) are currently in Phase I.

Envafolimab is a single-domain PD-L1 antibody and administered subcutaneously. There is an ongoing phase II trial evaluating envafolimab alone and with ipilimumab in undifferentiated pleomorphic sarcoma or myxofibrosarcoma (ENVASARC, NCT04480502). A multicenter phase II trial of paclitaxel alone and with nivolumab in taxane-naïve angiosarcoma patients is ongoing. (Alliance A091902, NCT04339738).

In recent years, nanotechnology has shown potential in sarcoma treatment thanks to the development of smart materials and more effective drug delivery systems. Examples include effective docetaxel-loaded mPEG-PLA nanoparticles in sarcoma-bearing mice and albumin-paclitaxel (nab-paclitaxel/Abraxane™) in osteosarcoma mice (76–78). (79) Only four nano-drug delivery systems have been FDA-approved for sarcoma - Doxil (Caelyx)^®^ for AIDS-related Kaposi’s sarcoma, DaunoXome^®^ and Lipo-Dox^®^ for Kaposi’s sarcoma and Liposomal mifamurtide (MEPACT) for Osteosarcoma. For locally advanced STS, there was a randomized, controlled Phase II-III trial by Bonvalot et al. in 2019 which investigated the role of NBTXR3, a radiation-enhancing nano-particle with radiotherapy compared to radiotherapy alone, demonstrated the efficacy of NBTXR3 with radiation (CR 16% vs. 8%, p = 0.044). There already exists pre-clinical evidence in 2014 which demonstrated that the chitosan nanoparticle-Methylglyoxal complex has effective antitumor properties and elicits macrophage-mediated immunity in Sarcoma-180 tumor-bearing mice (80). A Phase I trial with BO-112 (a synthetic RNA conjugated with nano-sized polyethyleneimine, which activates the immune system) with nivolumab before surgery for resectable STS, is active since 2020. (NCT04420975)

The immunosuppressive microenvironment in STS should be easier to overcome with safer and more effective next-generation immunotherapy. It is currently understood that MMR deficiency is rare and tumor mutation burden is low (3.3/Mb) in STS (7, 81–84). In addition to a traditional concept of “immunologically hot” sarcoma with complex karyotypes which expresses high immune-infiltrate TME and responds well to immunotherapy, there is emerging evidence of epigenetic modulation of transcription in sarcoma, which boosts immunogenicity (85, 86). In a retrospective study of 35 patients, DNA methylation degree correlated with response to anti-PD-1 therapy in sarcoma (87).

There remains a question of whether the mutational burden or neoantigen in STS is clinically correlated to treatment response in immunotherapy. Tumor-infiltrating lymphocytes and PD-L1 expression in STS have shown conflicting prognostic significance thus far. Advancements in bioinformatics and molecular technology will guide the finding of potential biomarkers, which will help fine-tune more effective combinations for each patient in future trials.

## 6 Summary

Advanced soft tissue sarcoma is still a devastating diagnosis, and there are limited treatments that have long-term success rates.

This article reviewed current immunotherapy in STS, mainly immune checkpoint inhibitors alone or with additional local/systemic therapy and adoptive cell therapy, which modifies the immunogenicity of tumors and TME.

There is a dire need to identify genetic and clinical indicators of response, resistance, and toxicity in immunotherapy in STS. To better characterize histologic/molecular subtypes of STS, tissue and liquid biopsies should be more frequently utilized.

Advancement in the laboratory and clinical immunotherapy of STS for the last five years has been encouraging. By learning from each patient in clinical trials, we hope that patients with soft tissue sarcoma can benefit in the new era of immunotherapy.

## Author contributions

GS writing - original draft and editing. SD conceptualization, review, and supervision. All authors contributed to the article and approved the submitted version.
